# Supplementary material for: Deep brain stimulation of the subgenual cingulum and uncinate fasciculus for the treatment of posttraumatic stress disorder
Source: Sci Adv. 2022 Dec 2;8(48):eadc9970. doi: 10.1126/sciadv.adc9970 (PMC10936049; doi:10.1126/sciadv.adc9970)
Supplement: 20221202-1 [file sciadv.adc9970.v1.pdf]

NEUROSCIENCE

Copyright © 2022 The Authors, some rights reserved; exclusive licensee American Association for the Advancement of Science. No claim to original U.S. Government Works. Distributed under a Creative Commons Attribution License 4.0 (CC BY).

Deep brain stimulation of the subgenual cingulum and uncinate fasciculus for the treatment of posttraumatic stress disorder

Clement Hamani[1,2,3]*, Benjamin Davidson[2,3], Felipe Corchs[4], Agessandro Abrahao[1,2,5], Sean M. Nestor[1,2,6], Jennifer S. Rabin[1,2,5,7], Alexander J. Nyman[1,2], Liane Phung[1,2], Maged Goubran[1,8], Anthony Levitt[1,6], Omid Talakoub[2], Peter Giacobbe[1,2,6], Nir Lipsman[1,2,3]

Deep brain stimulation (DBS) has been investigated for neuropsychiatric disorders. In this phase 1 trial, we treated four posttraumatic stress disorder (PTSD) patients with DBS delivered to the subgenual cingulum and the uncinate fasciculus. In addition to validated clinical scales, patients underwent neuroimaging studies and psychophysiological assessments of fear conditioning, extinction, and recall. We show that the procedure is safe and potentially effective (55% reduction in Clinical Administered PTSD Scale scores). Posttreatment imaging data revealed metabolic activity changes in PTSD neurocircuits. During psychophysiological assessments, patients with PTSD had higher skin conductance responses when tested for recall compared to healthy controls. After DBS, this objectively measured variable was significantly reduced. Last, we found that a ratio between recall of extinguished and nonextinguished conditioned responses had a strong correlation with clinical outcome. As this variable was recorded at baseline, it may comprise a potential biomarker of treatment response.

INTRODUCTION

It is estimated that approximately 50 to 60% of the American population will be exposed to at least one lifetime traumatic event (*1*–*4*). Although most individuals recover from the experience, around 10% develop posttraumatic stress disorder (PTSD). While medications and psychotherapy are often effective, 20 to 30% of patients do not respond to conventional treatments (*5*). Deep brain stimulation (DBS) is an established therapy for movement disorders, such as Parkinson's disease and tremor. In psychiatry, this therapy is approved for the treatment of obsessive-compulsive disorder and is under investigation for depression, drug addiction, eating disorders, and Alzheimer's disease (*6*–*16*).

Preclinical experiments using electrical stimulation in rodent models of PTSD-like behavior have been conducted to investigate the neural circuits, cellular elements, and mechanisms involved in fear conditioning/extinction and anxiety (*17*–*26*). We have recently found that chronic stimulation of the ventromedial prefrontal cortex (vmPFC) of rats presenting a PTSD-like phenotype significantly improved abnormal fear and anxiety responses, countered dysfunctional circuit connectivity, and reduced firing of basolateral amygdala (BLA) principal cells (*27*–*29*).

In clinical trials, DBS in a region homologous to the vmPFC [i.e., the subgenual cingulum (SCG)] has been investigated in patients with depression (*8*, *30*, *31*) and anorexia nervosa (*13*, *32*). To date, two patients with PTSD receiving DBS have been reported in the literature, both presenting a substantial postoperative improvement. The first was implanted with electrodes in the amygdala (*33*) and the second in the SCG, near the uncinate fasciculus (UF), a fiber tract that connects the PFC and the amygdala (*34*). This latter patient was the first treated in our center with the translational rationale of delivering DBS to modulate activity in the amygdala and reduce PTSD symptoms. Since our initial publication, three additional patients have been included in our trial.

We report the clinical outcome of four patients with PTSD treated with DBS in the region of the SCG/UF, including neuroimaging and psychophysiological fear conditioning, extinction, and recall assessments. Our data suggest that not only patients present a substantial clinical improvement but also the retention of fear extinction may predict treatment response.

RESULTS

Participants, surgical procedure, and safety

Four female patients were enrolled in this pilot trial (Table 1). The mean age at the time of surgery was 35.8 ± 3.8 years. All patients had severe PTSD, as measured by average Clinical Administered PTSD Scale (CAPS) scores of 77.5 ± 2.5. The mean duration of illness before DBS was 13.0 ± 1.4 years. All patients had comorbid depression and two had generalized anxiety disorder. The four patients suffered long-term sexual and/or verbal abuse along with additional traumatic experiences. Subjects did not respond to an average of six adequate courses of treatment (Table 1).

In our study, the implanted electrodes were directional. The most ventral and dorsal contacts were nondirectional (only to be used in a ring mode, that is, stimulation delivered from all its surface). The two middle rings could be subdivided in three independently activated contacts. Leads were implanted on the basis of tractography, so that one directional contact was placed near the UF, and an adjacent ring contact tentatively positioned in the crossroad between the cingulate bundle, UF, and forceps minor (Fig. 1) (*35*–*37*). As

[1]Sunnybrook Research Institute, Toronto, ON M4N3M5, Canada. [2]Harquail Centre for Neuromodulation, Sunnybrook Health Sciences Centre, Toronto, ON M4N 3M5, Canada. [3]Division of Neurosurgery, Sunnybrook Health Sciences Centre, University of Toronto, Toronto, ON M4N 3M5, Canada. [4]Department of Psychiatry, Institute of Psychiatry, University of São Paulo, SP 05403-903, Brazil. [5]Division of Neurology, Department of Medicine, Sunnybrook Health Sciences Centre, University of Toronto, ON M4N 3M5, Canada. [6]Department of Psychiatry, Sunnybrook Health Sciences Centre, University of Toronto, Toronto, ON M4N 3M5, Canada. [7]Rehabilitation Sciences Institute, University of Toronto, Toronto, ON M5G 1V7, Canada. [8]Department of Medical Biophysics, University of Toronto, Toronto, ON, Canada.
*Corresponding author. Email: clement.hamani@sunnybrook.ca

Table 1. Demographics. CAPS, clinician administered PTSD scale; CBT, cognitive behavioral therapy; CPT, cognitive processing therapy; DBT, dialectic behavior therapy; ECT, electroconvulsive therapy; EFT, emotion focused therapy; EMDR, eye movement desensitization and reprocessing; ERP, exposure and response prevention; GAD, generalized anxiety disorder; MDD, major depressive disorder; pt, patient; SNRI, serotonin and norepinephrine reuptake inhibitors; SSRI, selective serotonin reuptake inhibitors.

	CAPS	Major traumatic experience	Psychiatric comorbidities	Failed psychiatric treatments
Pt 1	72	Domestic abuse and death of daughter by motor vehicle accident	MDD and GAD	SSRI, SNRI, tricyclics, and mood stabilizers; CBT, CPT, EFT, and EMDR
Pt 2	80	Physical and sexual abuse	MDD	SSRI, SNRI, tricyclics beta-blockers, and anxiolytics; CBT, EMDR, and exposure therapy
Pt 3	75	Childhood emotional and physical abuse	MDD and GAD	SSRI, SNRI, tricyclics, ERP, EMDR, neurofeedback, and ECT
Pt 4	83	Childhood emotional, physical, and sexual abuse	MDD	SSRI, SNRI, antipsychotics, and anxiolytics; DBT, CBT, and group and individual therapies

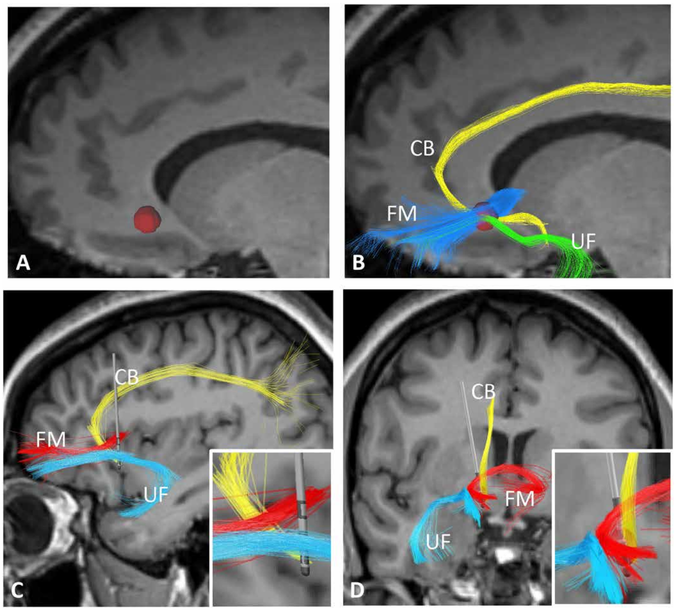

Fig. 1. Electrode placement. Targeting was based on the direct visualization of the SCG and diffusion tensor imaging (DTI). After anatomical visualization of the SCG at the gray-white junction below the frontal horn of the lateral ventricle in magnetic resonance imaging (MRI) slices, a 3.5-mm region of interest (ROI; red sphere) was used as a seed (**A**) to perform tractography and identify the cingulate bundle (CB), uncinate fasciculus (UF), and forceps minor (FM). The ROI was manually adjusted in the medial-lateral (x), anterior-posterior (y), and superior-inferior (z) dimensions to maximize the number of streamlines propagating through the UF (**B**). The final ROI was then used as a targeting site in a neurosurgical planning station. In the postoperative period, sagittal (**C**) and coronal (**D**) images were merged to preoperative MRI and DTI scans. Reconstructed electrodes show ring contacts near the SCG fiber blueprint and directional contacts near the uncinate fascicle. Squares in the right lower corner of (C) and (D) display magnified reconstructed images of the electrode amidst the fiber pathways described above.

our patients had comorbid depression, this approach was designed to stimulate this tract blueprint, which was previously associated with a good postoperative response (*35*, *36*), while steering current to the UF. Under these circumstances, we would stimulate the neurocircuitry of depression and, following our preclinical work, potentially modulate activity in the amygdala to improve PTSD symptoms (*29*).

Surgery was generally uneventful. Only one patient (#4) presented a severe adverse event unrelated to DBS. On postoperative month 4, she had appendicitis, followed by peritonitis. As a result, her 6-month follow-up evaluation was delayed by 1 month. Patient 1 had transient paresthesias in the region of the cranial incision that subsided on postoperative month 3. Patient 3 presented discomfort in the region of the connector between the electrodes and extension cables that was controlled with analgesics.

Programming sessions began 2 weeks after surgery and were conducted weekly until the third month, followed by biweekly sessions, when necessary. On the basis of our preclinical work (*29*) and clinical studies in depression (*31*, *36*, *38*), we have decided to use high-frequency stimulation (>100 Hz). Programming was conducted on the basis of tractography. Contacts in the ring implanted in the SCG were tested alone or in combination with the directional contact near the UF. Patients often had a more pronounced subjective improvement when the UF contact was activated in conjunction with SCG contacts. Current amplitude was increased or decreased according to subjective responses. Patient 4 was not responsive to stimulation, despite the activation of multiple contacts alone or in combination. At 6 months, cathodes in all patients were contacts implanted near the SCG fiber blueprint (ring contact in patients 3 and 4 or directional medial contacts in patients 1 and 2) and the UF (lateral directional contact in all subjects). In all patients, the case was used as anode, the frequency was set at 130 Hz, and the pulse width was set at 60 μs. Delivered amplitudes in patients 1 to 4 were 6.5, 2.0, 4.5, and 4 mA, respectively.

Outcome

Six months after DBS, CAPS scores were decreased by 55.4 ± 19.8% relative to baseline (Fig. 2). Of the four patients treated, two were responders (CAPS reduction, ≥50%), one had a partial response (CAPS reduction, ≥30%), and one was a nonresponder (CAPS reduction, <30%; table S1). Symptomatic amelioration was observed in all PTSD domains, as revealed by improvements in different CAPS criteria (Table 2). Average Short PTSD Rating Interview (SPRINT) 1 to 8 scores were improved by 53.7 ± 19.2% at 6 months. The perception of improvement at 6 months (SPRINT 9) was increased by 58.75 ± 19.2%. Symptoms were found to be much improved after treatment (SPRINT 10; 4.25 ± 0.5). Davidson Trauma Scale scores at 6 months were reduced by 54 ± 19.0% (Table 2).

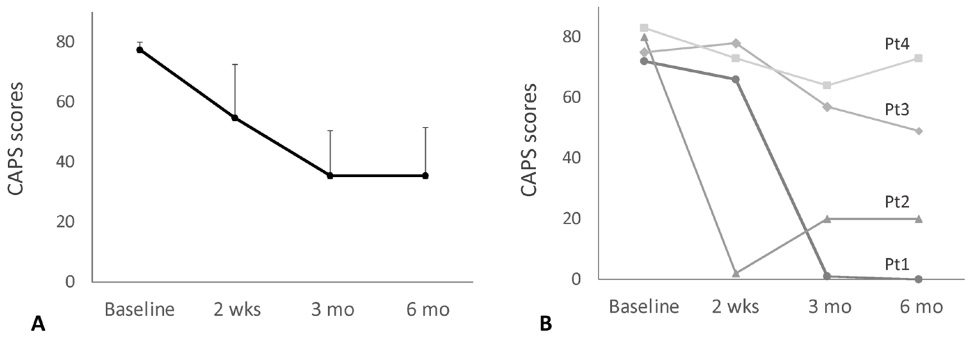

Fig. 2. Primary outcome data. (**A**) Average Clinician-Administered PTSD Scale (CAPS) scores were decreased by 55.4% 6 months (mo) after DBS relative to baseline. (**B**) Of the four patients (pt) included in our trial, two (#1 and #2) were considered to be responders (average CAPS improvement of 87.5%), one (#3) was a partial responder (35% improvement), and one (#4) was a nonresponder (12% improvement). Wks; weeks, mo, months.

Table 2. Clinical outcome. BAI, Beck's Anxiety Inventory; BDI, Beck's Depression Inventory; CAPS, Clinician-Administered PTSD Scale; CGI, Clinical Global Impression; DTS, Davidson Trauma Scale; GAF, Global Assessment of Functioning; HAMA, Hamilton Anxiety Rating Scale; HAMD, Hamilton Depression Rating Scale 17 items; improv, improvement; mo, months; SDS, Sheehan disability scale; SPRINT, Short PTSD Rating Interview; wk, week. Data represents means ± SE.

PTSD					
Scale	**Baseline**	**Wk 2**	**3 mo**	**6 mo**	**% improv. 6 mo**
CAPS	77.5 ± 2.5	54.75 ± 17.8	35.5 ± 15.0	35.5 ± 16.0	55.4 ± 19.8
Criterion B	20.25 ± 2.1	12.75 ± 4.6	8 ± 3.5	7.75 ± 3.2	60.0 ± 18
Criterion C	15.5 ± 1.8	9.5 ± 3.5	6 ± 2.7	6.25 ± 2.6	57.2 ± 20
Criterion D	4.75 ± 0.3	3.25 ± 1.1	2 ± 0.9	1.5 ± 0.6	67.5 ± 13.8
SPRINT 1–8	29.0 ± 1.1	22.5 ± 6.9	12.0 ± 6.0	14.0 ± 6.0	53.7 ± 19.2
SRPINT 9		22.5 ± 22.5	65.0 ± 15.5	58.75 ± 19.2	
SPRINT 10		2.75 ± 0.75	4.25 ± 0.48	4.25 ± 0.48	
DTS	105 ± 4.5	76.0 ± 21.4	46.8 ± 20.9	50.5 ± 21.3	54.0 ± 19.0
Depression anxiety					
Scale	**Baseline**	**Wk 2**	**3 mo**	**6 6 mo**	**% improv. 6 mo**
HAMD17	28 ± 2.6	20.25 ± 7.2	15.75 ± 5.4	18.75 ± 6.6	38.0 ± 18.5
BDI	40.5 ± 1.3	28.75 ± 9.7	16.75 ± 6.3	21.8 ± 7.8	47.8 ± 17.7
HAMA	32.5 ± 4.8	24.75 ± 8.8	19.75 ± 7.2	18 ± 6.3	47.5 ± 11.5
BAI	32.5 ± 5.6	28.75 ± 10.1	23.25 ± 7.6	22.25 ± 5.4	30.1 ± 11.8
GCI/GAF					
Scale	**Baseline**	**Wk 2**	**3 mo**	**6 mo**	**% improv. 6 mo**
CGI severity	6.25 ± 0.25	5.25 ± 1.1	4.0 ± 0.9	4.0 ± 1.1	36.9 ± 16.0
GAF	40.5 ± 3.8	50.5 ± 1.03	65.25 ± 8.5	63.75 ± 9.0	−55.5 ± 11.2
Disability					
SDS	**Baseline**	**Wk 2**	**3 mo**	**6 mo**	**% improv. 6 mo**
Combined	29.9 ± 0.4	22.0 ± 7.0	18.3 ± 5.2	17.0 ± 4.8	41.6 ± 16.5
Work	10.0 ± 0	7.8 ± 2.3	6.5 ± 1.8	6.3 ± 1.3	37.5 ± 12.8
Social	9.5 ± 0.3	7.3 ± 2.4	6.0 ± 2.0	5.5 ± 1.9	41.9 ± 21.4
Family/home	9.5 ± 0.3	7.0 ± 2.3	5.8 ± 1.7	5.3 ± 1.8	45.8 ± 18.2
Days lost	5.3 ± 1.75	3.5 ± 2.0	3.0 ± 1.8	3.5 ± 2.0	25.0 ± 25.0
Days unproductive	6.5 ± 0.5	4.5 ± 1.6	3.5 ± 1.6	4.0 ± 1.6	38.6 ± 22.1

In the preoperative period, two patients had severe, and two patients had very severe depression (table S2). Six months after DBS, improvements in Hamilton Depression Rating Scale 17 items (HAMD) and Beck's Depression Inventory (BDI) scores were of 38.0 ± 18.5% and 47.8 ± 17.6%, respectively (Table 2). The two PTSD responders had a ≥50% reduction in HAMD scores. In the partial responder, depression scores were reduced by 30% (table S2). Mean Hamilton Anxiety Rating Scale (HAMA) and Beck's Anxiety Inventory (BAI) scores were improved by 47.5 ± 11.5% and 30.1 ± 11.8%, respectively (Table 2).

Average Global Assessment of Functioning (GAF) and Clinical Global Impression (CGI) scores 6 months after surgery were improved by 55.5 ± 11.2% and 36.9 ± 16.0%, respectively (Table 2). According to the latter, patient 1 was very much improved (CGI 1), patient 2 was much improved (CGI 2), patient 3 was minimally improved (CGI 3), and patient 4 was minimally worse (CGI 5). Total Sheehan Disability Scores at 6 months were reduced by 41.6 ± 16.5% on average compared to baseline (Table 2). Medications were largely unchanged during the trial. There were no significant postoperative changes in any of the cognitive domains examined on neuropsychological testing (table S3).

Neuroimaging

Fluorodeoxyglucose positron emission tomography (^{18}F-FDG PET) was conducted at baseline and 6 months following DBS onset with the electrodes turned on. Patient #2 lived outside the province. Because of coronavirus disease 2019–related travel restrictions, her PET scan and psychophysiological testing were conducted 12 months after surgery.

Because of the small number of patients included in the study, we have limited our neuroimaging analyses to some regions of interest (ROIs) that either project or receive projections from the SCG (*39*) and are considered to be part of the neurocircuitry of PTSD (*26*, *40*). These included the amygdala, hippocampus, nucleus accumbens, cingulate cortex, and frontal cortical regions. Following DBS, patients had a significant increase in metabolic activity in the left amygdala (corrected for false discovery rate, *P*-FDR = 0.02) and right cingulate cortex (*P*-FDR = 0.001) compared to baseline, after correcting for age using a linear mixed effect model (Fig. 3). No significant postoperative changes were found in the remainder ROIs (table S4). An exploratory surface-based analysis did not reveal any significant changes in other cortical regions.

Psychophysiological testing

Psychophysiological testing to study fear conditioning and extinction was conducted over two consecutive days (*41*, *42*). Skin conductance responses (SCRs) were recorded via electrodes attached to the index finger of the nondominant hand. Electric shocks (annoying but not painful) were administered to the index finger of the dominant hand. During an initial habituation phase, images of an office with a table lamp were presented to participants (*41*). This was followed by a fear conditioning phase in which images of the same lamp shining a red or a blue light were shown during eight trials (Fig. 4A and fig. S1) (*41*). In five of these trials, image presentation was followed by the delivery of a 0.5-s electric shock (conditioned stimulus; CS+) (*41*). The third colored lamp (yellow) was presented 16 times with no associated shocks (nonconditioned stimulus; CS−; Fig. 4A). Five minutes later, subjects underwent a fear extinction phase, during which they looked at images containing similar lamps in a different context (a library instead of an office; fig. S1). The CS+ red light was presented 16 times with no electric shock pairing, intermixed with 16 presentations of the CS− (yellow light). On the following day, participants were exposed to extinction memory recall testing. Both extinguished (CS+ E; red light) and nonextinguished stimuli (CS+ NE; blue light) were presented eight times each, along with 16 presentations of the CS− (*41*). No shocks were delivered during recall.

Data were analyzed by comparing pre- and postoperative findings in the four patients with PTSD and three age/sex matched controls. Overall, similar preoperative SCR values were recorded during the acquisition (fear conditioning) and extinction phases. In contrast, a trend toward higher SCR responses was observed in patients with PTSD during the recall of CS+ E (*P* = 0.057) but not CS+ NE stimuli (Fig. 4, B and C). In the postoperative follow-up visit, objective SCR measures during recall of CS+ E (but not CS+ NE) were significantly reduced in patients with PTSD (Fig. 5, A and B), reaching levels similar to those observed in controls (Fig. 5, C and D).

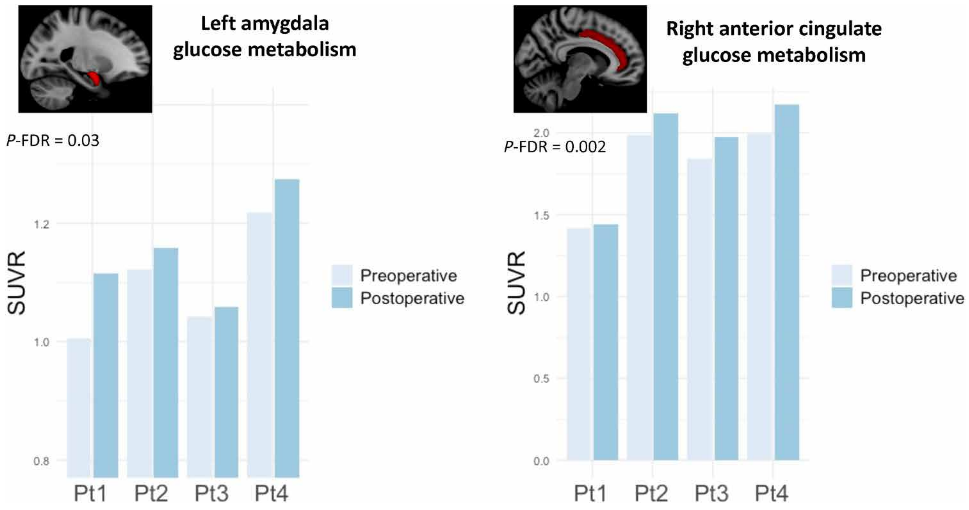

Fig. 3. Fluorodeoxyglucose positron emission tomography. Following DBS, patients had a significant increase in metabolic activity in left amygdala and right anterior cingulate cortex compared to baseline. These results were consistent in all four patients of the trial. SUVR, standardized uptake value ratio images.

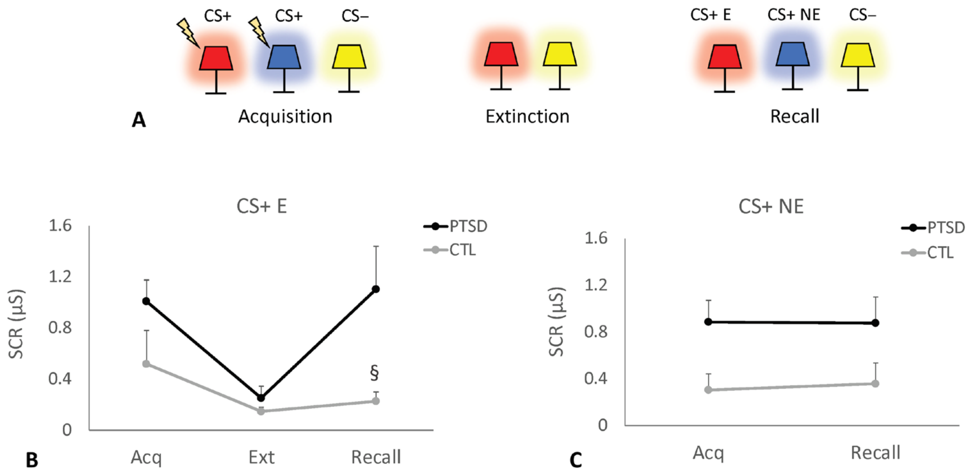

Fig. 4. Psychophysiological testing at baseline. (**A**) During the acquisition (Acq) of fear conditioning, images of a lamp shining a red, blue, or yellow light were shown. The former two (conditioned stimuli; CS+) but not the latter (unconditioned stimuli; CS−) were accompanied by an electric shock. SCR was measured during the trials. During fear extinction (Ext), the CS+ red light and CS− yellow light were presented in the absence of electric shocks. On the following day, participants were exposed to extinction memory recall testing. Nonextinguished stimuli (CS+ NE; blue light), extinguished stimuli (CS+ E; red light), and CS− (yellow light) were shown to the patients and controls (CTL) with no shock pairing. (**B**) Similar preoperative SCR values were recorded during the acquisition and extinction phases. In contrast, a trend toward higher SCR responses was observed in patients with PTSD during the recall of CS+ E ($P = 0.057$). (**C**) SCR measurements during the acquisition and recall of CS+ NE stimuli were similar in patients with PTSD and controls at baseline. µS, microsiemens; §, trend toward significance.

Because differences between patients with PTSD and controls were largely observed in the recall phase, we have decided to analyze the extinction retention index, which takes into account the relation between SCR during recall and the acquisition phase (100 − ([recall value/acquisition value] × 100) (*42*). In general, high values denote a situation in which SCR during recall is lower than in acquisition, meaning that the subject extinguished the conditioned stimuli. Overall, differences between groups were not found to be significant, despite lower values being recorded in patients relative to controls (fig. S2).

As SCR values varied substantially across both PTSD and control individuals, we have decided to study the relation between the extinction retention index during CS+ E and CS+ NE stimuli as a ratio: CS+ E/CS+ NE × 100. While no significant differences were recorded, we noticed that controls had a higher retention of CS+ E compared to CS+ NE, whereas the opposite results were found in patients with PTSD (fig. S2). Notably, a strong correlation was found between the percent improvement in CAPS scores and the ratio described above, either at baseline ($r = -0.86$) or during the postoperative period ($r = 0.70$). This suggests that patients who improved the most after surgery had the highest retention of extinction of CS+ E relative to CS+ NE.

DISCUSSION

Despite the proposal of a clinical trial (*43*), before our series, only two case reports have been published using DBS to treat PTSD: our previously published patient (*34*) and a combat veteran who had a 37.8% postoperative improvement in CAPS scores following amygdala stimulation (*33*). In our current trial, four females with a long-term history of sexual and/or verbal abuse and acute traumatic events were recruited. Six months after DBS, average CAPS scores were decreased by 55% compared to baseline. Of the four patients, two responded to the procedure (average CAPS improvement of 87.5%), one was a partial responder (35% improvement in CAPS scores), and one was a nonresponder. In our series, symptoms in all PTSD domains improved to a similar extent. In addition to efficacy, SCG/UF DBS was found to be safe, with a profile of side effects comparable to the one reported in other DBS trials for psychiatric disorders (*8*, *32*, *38*).

In general, 40 to 55% of patients undergoing a traumatic event present a spontaneous recovery (*5*, *44*, *45*). Forecasting a poor prognosis is childhood abuse (*45*), which occurred in the four patients of our trial. Treatments for chronic PTSD include pharmacotherapy and psychotherapy. In general, it is estimated that 30% of patients will fail these interventions and may ultimately be labeled as treatment refractory (a term that lacks a precise definition) (*5*). In general, patients who do not respond to conventional treatments have more complex and protracted forms of the disease, associated comorbidities (e.g., treatment refractory depression) and multiple traumatic experiences. Despite the relatively high percentage of patients with refractory PTSD, we have only received four referrals. After discussing the potential risks and benefits of DBS for other psychiatric indications, the four patients immediately agreed to sign the informed consent, stating that their illness was very severe and that they were extremely debilitated.

Our choice of target was based on translational preclinical and clinical work. We have recently shown that chronic vmPFC DBS significantly improved anxiety and extinction recall in a rodent model of PTSD (*29*). In animals presenting a behavioral PTSD-like phenotype, DBS induced a significant reduction in BLA principal cell firing and an increase in interneuron activity (*29*). Considering the potential top-down inhibition of the amygdala, we have decided to stimulate the SCG in the vicinity of the UF. Since all enrolled patients had depression, we implanted electrodes with one ring contact in the crossroad between the cingulate bundle, UF, and forceps minor (*35*–*37*). During programming, patients preferred a combined stimulation approach (SCG + UF) rather than SCG stimulation alone. Since our trial was preliminary in nature, however, it did not allow more assertive conclusions on the role of each fiber

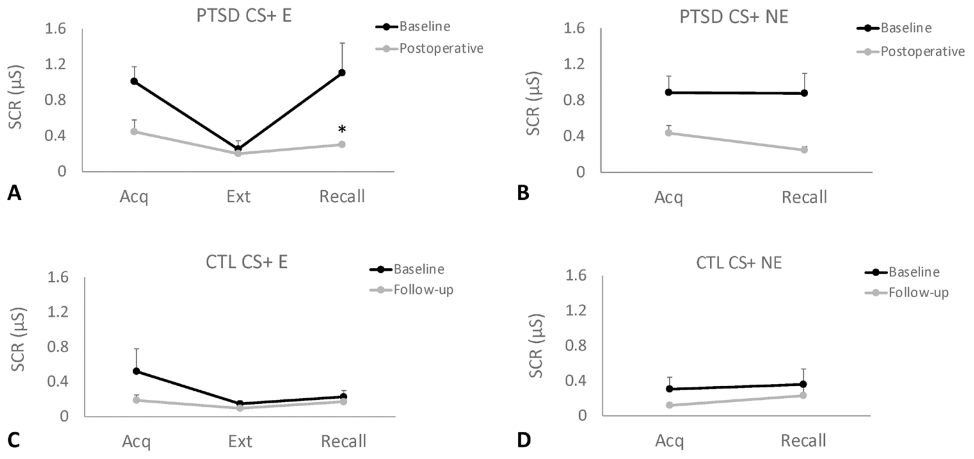

Fig. 5. Psychophysiological testing in the preoperative and postoperative periods. (**A** and **B**) SCRs recorded in patients with PTSD were similar in the acquisition and extinction phases at baseline and in the postoperative period. During the recall of extinguished stimuli (CS+ E), but not the recall of nonextinguished stimuli (CS+ NE), patients with PTSD treated with DBS had a significant decrease ($P = 0.03$) in SCR values compared to baseline, with values reaching levels similar to those observed in age/sex matched controls (CTL; **C** and **D**). Asterisk (*) indicates significant differences.

tract in a DBS response. Additional systematic studies are certainly necessary to address this issue.

Of all regions analyzed in our study, significant pre- and postoperative differences were only found in the left amygdala and the right anterior cingulate cortex. On the basis of our preclinical data, we hypothesize that the augmented amygdala metabolism following DBS could be due to an increased interneuron activity, which would subsequently reduce principal cell firing (*29*). As for the cingulum, it is possible that the increased metabolism observed in the postoperative period may reflect an activation of the cingulate bundle following DBS. In our study, postoperative PET scans were acquired while patients were receiving stimulation. Under these circumstances, it is difficult to disentangle nonspecific effects of DBS from those associated with a clinical improvement. This, however, would have been difficult to accomplish even if the electrodes were turned off before the scans. Overall, DBS is known to induce neuroplasticity and has an important carryover effect upon stimulation offset (*26*, *46*, *47*). As patients had different clinical outcomes despite relatively similar PET results, we hypothesize that metabolic changes in our study were predominantly associated with a general DBS response. As a result, we could not differentiate responders versus nonresponders based on PET data.

An intriguing aspect of our trial was the unilateral nature of PET changes. Over the years, several neuroimaging modalities have been used to study volumetric and activation patterns in PTSD, particularly in the amygdala (*48*–*50*). Overall, results have not been homogeneous, with changes being recorded unilaterally (right or left) and bilaterally (*48*, *50*, *51*). In some of these studies, patients with PTSD were shown to have a volumetric reduction in the left amygdala compared to trauma-exposed controls (*52*, *53*) or psychiatric patients with other diagnoses (*54*). Veterans with PTSD had stronger oscillatory activity in the amygdala when exposed to threatening faces compared to veterans without PTSD (*55*). In a psychotherapy study, the attenuation of functional interactions, including those from the left PFC to the left amygdala, was associated with symptom reduction (*56*). At present, we cannot explain why metabolic changes following DBS were only observed in the left amygdala. As electrodes were placed symmetrically in both hemispheres, currents delivered to most patients were in the range of 4 to 6.5 mA, and the metabolic pattern observed in our study was equally observed in all patients, it is unlikely that differences in the volume of tissue activated may explain our unilateral findings. Future studies will be necessary to corroborate and explain the hemispheric differences observed in our study.

Psychophysiological data in our trial were acquired and analyzed according to a previously used protocol (*41*, *42*). Our study corroborates previous work in the field suggesting that patients with PTSD have an impaired extinction recall but no significant differences in skin conductance during acquisition or extinction compared to trauma-exposed controls (*57*). To test whether neuromodulation strategies could improve extinction learning, healthy individuals received transcranial magnetic stimulation paired to a conditioned cue during extinction (*58*). Subjects not only had a reduced SCR but also changes in distinct cortical regions and large-scale fear networks (*58*). In our study, patients were tested before and after DBS. Because test-retest results in this protocol have not been well characterized, we have decided to study an age/sex matched control group as well. Similar to previous work (*57*), no SCR differences were found between patients with PTSD and controls during the acquisition and extinction phases of the test. The former group, however, showed a strong trend toward higher SCRs during the recall of extinguished stimuli. This was significantly improved in the postoperative period, with SCRs in patients with PTSD reaching levels comparable to those observed in controls.

One aspect that became clear during the study was that skin conductance values varied substantially across individuals in both patients with PTSD and controls. It has been previously demonstrated that patients with PTSD have an increased expectation to unconditioned stimuli in general or following CS− compared to healthy controls (*59*). This was verbally reported by patients in our trial. As a result, we have decided to study the relation between the extinction retention index recorded when patients were exposed to extinguished and nonextinguished stimuli. We found that this ratio had a strong correlation with the percentage of postoperative CAPS improvement. Overall, patients who improved the most after surgery had the highest extinction retention of CS+ E relative to CS+ NE at baseline. Although

future studies are needed to corroborate these findings, our results suggest that the ratio described above may be a potential biomarker of treatment response in patients with PTSD undergoing DBS.

Our study has a few limitations that need to be discussed, particularly in relation to the small sample size. As 30% of patients with PTSD are considered to be treatment refractory, we estimated that at least 10% of patients in high-volume centers would have failed multiple therapeutic modalities, including electroconvulsive therapy, different medications, psychotherapy, or even off-label and investigative approaches. These numbers should have generated a larger referral basis compared to the few individuals sent to our clinic. We hope the positive safety and efficacy results of our study may encourage psychiatrists to refer patients for future trials, particularly if one considers the tremendous suffering and poor quality of life associated with refractory forms of the disorder.

In the first conception of our trial, we thought that ideal candidates would have been patients suffering acute traumatic experiences that developed PTSD and did not improve with medications and/or psychotherapy. These subjects, however, often respond to conventional treatment, which limits the number of surgical candidates to patients with associated comorbid diagnoses and more protracted and complex forms of the disease (*60*–*62*).

Patients in our trial had PTSD and comorbid depression. Although improvements in depression could have contributed to our overall results, the amelioration in PTSD symptoms observed in validated scales (CAPS, SPRINT, and Davidson Trauma Scale) and the objective reduction in SCR during recall sessions suggest that the beneficial effects of DBS were, at least in part, associated with improvements in extinction memory recall and PTSD symptoms.

In summary, we showed that DBS delivered to the SCG and UF is safe and effective in patients with PTSD. In addition to clinical aspects and metabolic changes in the circuitry of fear and anxiety, our results suggest that psychophysiological metrics can potentially be used as a biomarker of treatment response and help to delineate objective outcome measures. Future studies in a larger number of patients are required to corroborate our preliminary findings.

MATERIALS AND METHODS

The study was approved by the Research Ethics Board of Sunnybrook Health Sciences Centre and registered in ClinicalTrial.gov (NCT03416894). Before enrolling, participants provided written informed consent. Patients were referred by their primary psychiatrists to the Harquail Centre for Neuromodulation.

Inclusion and exclusion criteria

Inclusion criteria are as follows: (i) females or males between the ages of 18 and 70; (ii) diagnosis of PTSD, as defined by the *Diagnostic and Statistical Manual fifth edition*; (iii) treatment resistance, characterized by the persistence of clinical symptoms despite adequate treatment with four therapeutic modalities, including (a) selective serotonin reuptake inhibitors, (b) cognitive behavioral therapy, and (c) other classes of medications and/or psychotherapy; (iv) severe forms of the disease, as measured by CAPS scores ≥50; (v) a pattern of chronic stable PTSD lasting at least 1 year; and (vi) ability to provide informed consent and comply with all testing, follow-up appointments, and protocols.

Exclusion criteria are as follows: (i) any past or current evidence of psychosis or mania (patients with comorbid depression were not excluded); (ii) active neurologic disease; (iii) alcohol or substance dependence or abuse in the last 6 months, excluding caffeine and nicotine; (iv) current suicidal ideation; (v) any contraindication to magnetic resonance imaging (MRI) or PET scanning; (vi) likely to relocate or move out of the country; (vii) presence of clinical and/or neurological conditions that may significantly increase the risk of the surgical procedure; and (viii) currently pregnant (as determined by history and serum HCG) or lactating.

Baseline evaluations

Four patients were screened. All met inclusion criteria and agreed to participate in the trial. After signing the consent form, clinical, psychiatric, and neurosurgical evaluations were performed. Psychiatric symptoms, disease severity, and quality of life were assessed with the following scales: (i) *Clinician-Administered PTSD Scale for The Diagnosis and Statistical Manual of Mental Disorders fifth edition*, (ii) Hamilton Depression Rating Scale 17 items, (iii) Beck's Depression Inventory, (iv) Beck's Anxiety Inventory, (v) Hamilton Anxiety Rating Scale, (vi) Davidson Trauma Scale, (vii) Short PTSD Rating Interview, (viii) Clinical Global Impression, (ix) Global Assessment of Functioning, and (x) Sheehan disability scale. Side effects were recorded and evaluated with the Systematic Assessment for Treatment Emergent Events collateral effects scale. Before surgery, patients underwent neuropsychological assessments, MRI, ^{18}F-FDG PET, psychophysiological testing, and a preoperative anesthetic assessment.

Targeting and surgical procedure

Preoperative scans were acquired on a 3-T MRI (Magnetom Prisma; Siemens Healthcare, Germany). Anatomical images included a T1-weighted Magnetization Prepared–RApid Gradient Echo sequence with 192 slices (TE = 2.94 ms, TR = 2000 ms, matrix size = 256 × 256, resolution = 0.90 × 0.90 × 1.0 mm). Diffusion MRI scanning was performed using a total of 64 diffusion sampling directions (b value = 1000 s/mm^2), with an in-plane resolution and slice thickness of 2 mm. The diffusion data were reconstructed using generalized q sampling imaging with a diffusion sampling length ratio of 1.25 (*63*).

Surgery occurred within 1 month of the evaluations. Targeting was based on the direct visualization of the anatomical SCG and diffusion tensor imaging (DTI) showing projections from the PFC to the medial temporal lobe. We used a modified version of the targeting scheme described by Riva-Posse *et al.* (*35*–*37*). First, the SCG was targeted on a coronal slice at the gray-white junction, below the frontal horn of the lateral ventricle, as previously described (*64*). Next, DSI-Studio (http://dsi-studio.labsolver.org/) was used to rotate the DTI images and corresponding b-table to the space of the T1 scan. A 3.5-mm ROI was used as a seed to perform tractography and identify the blueprint consisting of the cingulum, UF, and forceps minor (*36*). The ROI was manually adjusted in the medial-lateral (x), anterior-posterior (y), and superior-inferior (z) dimensions, to maximize the number of streamlines propagating through the cingulum and the UF. The final ROIs were transferred to the Stealth Planning Station. The selected trajectory was intended to have the middle two contacts centered within the ROI while avoiding large vessels and sulci. A Cosman-Roberts-Wells frame was placed under local anesthesia and sedation, as previously described (*34*). The implanted DBS electrodes (Vercise Cartesia Directional Lead, Boston Scietific) have eight contacts. The most dorsal and ventral rings are nondirectional. The two middle ones may be subdivided in three equal contacts. In our study, electrodes were implanted so that one

directional ring was placed near the UF, while an adjacent contact was placed in the tract blueprint described above. Under these circumstances, contacts in one ring would stimulate the blueprint and a directional contact in the adjacent ring would steer current to the UF. Extension cables and a pulse generator (Vercise, Boston Scietific) were implanted during the same operating procedure under general anesthesia. Electrode placement was confirmed with a postoperative thin-slice computed tomography scan, which was used to localize and reconstruct electrode locations using Lead-DBS v2.0 software (www.lead-dbs.org/) (*65*). Postoperative images were registered to preoperative structural T1 MRI using SPM12 (www.fil.ion.ucl.ac.uk/spm/software/spm12/), followed by nonlinear registration to Montreal Neurologic Institute space (ICBM 2009b NLIN asymmetric) with Advanced Normalization Tools (*66*). After a semi-automated electrode localization process, electrodes were manually localized. Volumes of tissue activated were estimated using the FieldTrip-SimBio finite element method (www.mrt.uni-jena.de/simbio/index.php/; http://fieldtriptoolbox.org) as implemented through Lead-DBS.

Postoperative evaluations

Scales to measure psychiatric symptoms, disease severity, and quality of life were recorded 2 weeks, 3 months, and 6 months after the procedure. Neuropsychological assessments, psychophysiological testing, and ^{18}F-FDG PET were obtained at the sixth month follow-up, except for patient 2, as described above.

Neuropsychology

Evaluations consisted of a battery to test a broad range of cognitive domains, including processing speed, executive function, memory, and language. The following tests were applied: Brief Visuospatial Memory Test, California Verbal Learning Test, Delis-Kaplan Executive Function System, Frontal Systems Behavior Scale, Symbol Digit Modalities Test, and Wechsler Test of Adult Reading. To minimize practice effects, alternate versions of the tests were used at follow-up whenever possible. Pre- and postoperative test scores were compared with the Wilcoxon signed-rank test.

Fluorodeoxyglucose positron emission tomography

A transmission scan followed by a 20-min emission scan (four frames/5 min each) was acquired on a Phillips Gemini PET computed tomography (three-dimensional mode) 30 min after the administration of 5 mCi ± 10% of ^{18}F-FDG radiotracer. All frames were corrected for attenuation, detector deadtime, scatter, and radioisotope decay, coregistered and averaged to create a single frame (*66*). Scans corresponding to their FreeSurfer-processed T1 images were processed using the default settings of PETsurfer pipeline (https://surfer.nmr.mgh.harvard.edu/fswiki/PetSurfer). The steps consisted of (i) creating a high-resolution segmentation for subsequent partial volume correction (PVC) using the cortical and subcortical structures from the FreeSurfer cross-sectional pipeline, (ii) registration of the average PET image with the respective FreeSurfer T1 time point, and (iii) applying PVC to each registered PET image, normalized by standardized uptake values in the pons, and smoothed using a 5-mm full width at half maximum kernel, creating baseline and posttreatment standardized uptake value ratio (SUVR) images for each subject (*67*, *68*). To compare metabolic changes from baseline to posttreatment, surface-based and subcortical analyses were carried out. For the surface-based (cortical) analysis, a general mixed model (in this case, a paired t test) analysis was performed in FreeSurfer using age as a regressor of no interest. To correct for multiple comparisons, a Monte Carlo simulation with 5000 simulations was performed with a two-tailed cluster-wise P value < 0.05. A second model was constructed using clinical response at 6 months (% improvement on the CAPS) as a third regressor, which was then subjected to the same multiple comparison correction. For the subcortical analysis, the following ROIs were used from the Harvard-Oxford Subcortical Atlas (*69*): frontal pole, orbitofrontal cortex, anterior cingulate cortex, amygdala, nucleus accumbens, and hippocampus. Mean SUVR values were generated for each ROI at each time point and used to perform linear mixed models using time and age as regressors. An FDR correction was performed for each hemisphere. Mixed models that incorporated postoperative clinical response as a third regressor were also constructed.

Psychophysiological testing

The fear conditioning and extinction paradigm used in our study were conducted over two consecutive days, as previously described by others (*41*). For the recording of SCRs, two Ag/AgCl electrodes (9 mm in diameter) were attached to the index finger of the nondominant hand. Similar electrodes were placed on the finger of the dominant hand and used to deliver an electric shock. Before the experiments on day 1, participants had to choose a current threshold deemed to be annoying but not painful. During an initial habituation phase, images of an office with a table lamp were presented to participants (*41*). This was followed by a fear conditioning phase. Initially, a similar image was displayed for 3 s. Thereafter, images with the lamp shining a red or a blue light were presented eight times for 6 s each. In five of these trials, image presentation was followed by the delivery of a 0.5-s electric shock (CS+) (*41*). The third colored lamp (yellow) was presented 16 times with no associated shock (CS−). Five minutes later, subjects underwent a fear extinction phase, during which they looked at images containing similar lamps in a different context (a library instead of an office). The CS+ red light was presented 16 times with no electric shock pairing. Images were intermixed with 16 presentations of the CS− (yellow light; 6 s per image). On the following day, participants were exposed to extinction memory recall testing. Both extinguished and nonextinguished CS+ were presented eight times each, along with 16 presentations of the CS− (*41*). No shocks were delivered during recall. In all phases, stimuli were presented in a pseudorandom order. The intertrial intervals ranged from 12 to 18 s.

The acquisition value consisted of the average of the three largest SCR responses recorded during the acquisition phase. The extinction value was the average of the last two SCR extinction responses. Recall was the average SCR value recorded during the first two trials of extinction recall. The extinction retention index was calculated as described above. Differences between groups were compared with the Mann-Whitney test. Correlation analysis was used to assess the relationship between symptomatic improvement and psychophysiological scores.

SUPPLEMENTARY MATERIALS

Supplementary material for this article is available at https://science.org/doi/10.1126/sciadv.adc9970

REFERENCES AND NOTES

1. N. Breslau, G. C. Davis, P. Andreski, E. Peterson, Traumatic events and posttraumatic stress disorder in an urban population of young adults. *Arch. Gen. Psychiatry* **48**, 216–222 (1991).

2. N. Breslau, R. C. Kessler, H. D. Chilcoat, L. R. Schultz, G. C. Davis, P. Andreski, Trauma and posttraumatic stress disorder in the community: The 1996 Detroit area survey of trauma. *Arch. Gen. Psychiatry* **55**, 626–632 (1998).
3. J. A. Fairbank, L. Ebert, E. J. Costello, Epidemiology of traumatic events and post-traumatic stress disorder, in *Post Traumatic Stress Disorder: Diagnosis, Management and Treatment*, D. Nutt, J. R. T. Davidson, J. Zohar, Eds. (Martin Dunitz Ltd, London, 2000), pp. 17–27.
4. R. C. Kessler, A. Sonnega, E. Bromet, M. Hughes, C. B. Nelson, Posttraumatic stress disorder in the National Comorbidity Survey. *Arch. Gen. Psychiatry* **52**, 1048–1060 (1995).
5. R. J. Koek, H. N. Schwartz, S. Scully, J. P. Langevin, S. Spangler, A. Korotinsky, K. Jou, A. Leuchter, Treatment-refractory posttraumatic stress disorder (TRPTSD): A review and framework for the future. *Prog. Neuropsychopharmacol. Biol. Psychiatry* **70**, 170–218 (2016).
6. B. D. Greenberg, L. A. Gabriels, D. A. Malone Jr., A. R. Rezai, G. M. Friehs, M. S. Okun, N. A. Shapira, K. D. Foote, P. R. Cosyns, C. S. Kubu, P. F. Malloy, S. P. Salloway, J. E. Giftakis, M. T. Rise, A. G. Machado, K. B. Baker, P. H. Stypulkowski, W. K. Goodman, S. A. Rasmussen, B. J. Nuttin, Deep brain stimulation of the ventral internal capsule/ventral striatum for obsessive-compulsive disorder: Worldwide experience. *Mol. Psychiatry* **15**, 64–79 (2010).
7. D. A. Malone Jr., D. D. Dougherty, A. R. Rezai, L. L. Carpenter, G. M. Friehs, E. N. Eskandar, S. L. Rauch, S. A. Rasmussen, A. G. Machado, C. S. Kubu, A. R. Tyrka, L. H. Price, P. H. Stypulkowski, J. E. Giftakis, M. T. Rise, P. F. Malloy, S. P. Salloway, B. D. Greenberg, Deep brain stimulation of the ventral capsule/ventral striatum for treatment-resistant depression. *Biol. Psychiatry* **65**, 267–275 (2008).
8. A. M. Lozano, H. S. Mayberg, P. Giacobbe, C. Hamani, R. C. Craddock, S. H. Kennedy, Subcallosal cingulate gyrus deep brain stimulation for treatment-resistant depression. *Biol. Psychiatry* **64**, 461–467 (2008).
9. H. S. Mayberg, Modulating dysfunctional limbic-cortical circuits in depression: Towards development of brain-based algorithms for diagnosis and optimised treatment. *Br. Med. Bull.* **65**, 193–207 (2003).
10. B. Nuttin, P. Cosyns, H. Demeulemeester, J. Gybels, B. Meyerson, Electrical stimulation in anterior limbs of internal capsules in patients with obsessive-compulsive disorder. *Lancet* **354**, 1526 (1999).
11. L. Mallet, M. Polosan, N. Jaafari, N. Baup, M. L. Welter, D. Fontaine, S. T. du Montcel, J. Yelnik, I. Chereau, C. Arbus, S. Raoul, B. Aouizerate, P. Damier, S. Chabardes, V. Czernecki, C. Ardouin, M. O. Krebs, E. Bardinet, P. Chaynes, P. Burbaud, P. Cornu, P. Derost, T. Bougerol, B. Bataille, V. Mattei, D. Dormont, B. Devaux, M. Verin, J. L. Houeto, P. Pollak, A. L. Benabid, Y. Agid, P. Krack, B. Millet, A. Pelissolo, Subthalamic nucleus stimulation in severe obsessive-compulsive disorder. *N. Engl. J. Med.* **359**, 2121–2134 (2008).
12. A. M. Lozano, L. Fosdick, M. M. Chakravarty, J. M. Leoutsakos, C. Munro, E. Oh, K. E. Drake, C. H. Lyman, P. B. Rosenberg, W. S. Anderson, D. F. Tang-Wai, J. C. Pendergrass, S. Salloway, W. F. Asaad, F. A. Ponce, A. Burke, M. Sabbagh, D. A. Wolk, G. Baltuch, M. S. Okun, K. D. Foote, M. P. McAndrews, P. Giacobbe, S. D. Targum, C. G. Lyketsos, G. S. Smith, A phase II study of fornix deep brain stimulation in mild Alzheimer's disease. *J. Alzheimers Dis.* **54**, 777–787 (2016).
13. N. Lipsman, E. Lam, M. Volpini, K. Sutandar, R. Twose, P. Giacobbe, D. J. Sodums, G. S. Smith, D. B. Woodside, A. M. Lozano, Deep brain stimulation of the subcallosal cingulate for treatment-refractory anorexia nervosa: 1 year follow-up of an open-label trial. *Lancet Psychiatry* **4**, 285–294 (2017).
14. A. Goncalves-Ferreira, F. S. do Couto, A. Rainha Campos, L. P. Lucas Neto, D. Goncalves-Ferreira, J. Teixeira, Deep brain stimulation for refractory cocaine dependence. *Biol. Psychiatry* **79**, e87–e89 (2016).
15. R. Zhu, Y. Zhang, T. Wang, H. Wei, C. Zhang, D. Li, S. Zhan, B. Sun, Deep brain stimulation of nucleus accumbens with anterior capsulotomy for drug addiction: A case report. *Stereotact. Funct. Neurosurg.* **98**, 345–349 (2020).
16. B. Davidson, P. Giacobbe, T. P. George, S. M. Nestor, J. S. Rabin, M. Goubran, A. J. Nyman, A. Baskaran, Y. Meng, C. B. Pople, S. J. Graham, F. Tam, C. Hamani, N. Lipsman, Deep brain stimulation of the nucleus accumbens in the treatment of severe alcohol use disorder: A phase I pilot trial. *Mol. Psychiatry* 10.1038/s41380-022-01677-6, (2022).
17. M. R. Milad, I. Vidal-Gonzalez, G. J. Quirk, Electrical stimulation of medial prefrontal cortex reduces conditioned fear in a temporally specific manner. *Behav. Neurosci.* **118**, 389–394 (2004).
18. I. Vidal-Gonzalez, B. Vidal-Gonzalez, S. L. Rauch, G. J. Quirk, Microstimulation reveals opposing influences of prelimbic and infralimbic cortex on the expression of conditioned fear. *Learn. Mem.* **13**, 728–733 (2006).
19. J. P. Langevin, A. A. De Salles, H. P. Kosoyan, S. E. Krahl, Deep brain stimulation of the amygdala alleviates post-traumatic stress disorder symptoms in a rat model. *J. Psychiatr. Res.* **44**, 1241–1245 (2010).
20. G. J. Quirk, M. R. Milad, Neuroscience: Editing out fear. *Nature* **463**, 36–37 (2010).
21. G. J. Quirk, R. Garcia, F. Gonzalez-Lima, Prefrontal mechanisms in extinction of conditioned fear. *Biol. Psychiatry* **60**, 337–343 (2006).
22. G. J. Quirk, E. Likhtik, J. G. Pelletier, D. Pare, Stimulation of medial prefrontal cortex decreases the responsiveness of central amygdala output neurons. *J. Neurosci.* **23**, 8800–8807 (2003).
23. D. Sierra-Mercado, N. Padilla-Coreano, G. J. Quirk, Dissociable roles of prelimbic and infralimbic cortices, ventral hippocampus, and basolateral amygdala in the expression and extinction of conditioned fear. *Neuropsychopharmacology* **36**, 529–538 (2011).
24. R. Garcia, G. Spennato, L. Nilsson-Todd, J. L. Moreau, O. Deschaux, Hippocampal low-frequency stimulation and chronic mild stress similarly disrupt fear extinction memory in rats. *Neurobiol. Learn. Mem.* **89**, 560–566 (2008).
25. J. Rodriguez-Romaguera, F. H. Do Monte, G. J. Quirk, Deep brain stimulation of the ventral striatum enhances extinction of conditioned fear. *Proc. Natl. Acad. Sci. U.S.A.* **109**, 8764–8769 (2012).
26. R. Reznikov, M. Binko, J. N. Nobrega, C. Hamani, Deep brain stimulation in animal models of fear, anxiety, and posttraumatic stress disorder. *Neuropsychopharmacology* **41**, 2810–2817 (2016).
27. R. Reznikov, M. Diwan, J. N. Nobrega, C. Hamani, Towards a better preclinical model of PTSD: Characterizing animals with weak extinction, maladaptive stress responses and low plasma corticosterone. *J. Psychiatr. Res.* **61**, 158–165 (2015).
28. D. C. Gidyk, M. Diwan, F. V. Gouveia, P. Giacobbe, N. Lipsman, C. Hamani, Investigating the role of CB1 endocannabinoid transmission in the anti-fear and anxiolytic-like effects of ventromedial prefrontal cortex deep brain stimulation. *J. Psychiatr. Res.* **135**, 264–269 (2021).
29. R. Reznikov, F. R. Bambico, M. Diwan, R. J. Raymond, M. G. Nashed, J. N. Nobrega, C. Hamani, Prefrontal cortex deep brain stimulation improves fear and anxiety-like behavior and reduces basolateral amygdala activity in a preclinical model of posttraumatic stress disorder. *Neuropsychopharmacology* **43**, 1099–1106 (2018).
30. P. E. Holtzheimer, M. E. Kelley, R. E. Gross, M. M. Filkowski, S. J. Garlow, A. Barrocas, D. Wint, M. C. Craighead, J. Kozarsky, R. Chismar, J. L. Moreines, K. Mewes, P. R. Posse, D. A. Gutman, H. S. Mayberg, Subcallosal cingulate deep brain stimulation for treatment-resistant unipolar and bipolar depression. *Arch. Gen. Psychiatry* **69**, 150–158 (2012).
31. H. S. Mayberg, A. M. Lozano, V. Voon, H. E. McNeely, D. Seminowicz, C. Hamani, J. M. Schwalb, S. H. Kennedy, Deep brain stimulation for treatment-resistant depression. *Neuron* **45**, 651–660 (2005).
32. N. Lipsman, D. B. Woodside, P. Giacobbe, C. Hamani, J. C. Carter, S. J. Norwood, K. Sutandar, R. Staab, G. Elias, C. H. Lyman, G. S. Smith, A. M. Lozano, Subcallosal cingulate deep brain stimulation for treatment-refractory anorexia nervosa: A phase 1 pilot trial. *Lancet* **381**, 1361–1370 (2013).
33. J. P. Langevin, R. J. Koek, H. N. Schwartz, J. W. Chen, D. L. Sultzer, M. A. Mandelkern, A. D. Kulick, S. E. Krahl, Deep brain stimulation of the basolateral amygdala for treatment-refractory posttraumatic stress disorder. *Biol. Psychiatry* **79**, e82–e84 (2016).
34. C. Hamani, B. Davidson, A. Levitt, Y. Meng, F. Corchs, A. Abrahao, J. S. Rabin, P. Giacobbe, N. Lipsman, Patient with posttraumatic stress disorder successfully treated with deep brain stimulation of the medial prefrontal cortex and uncinate fasciculus. *Biol. Psychiatry* **88**, e57–e59 (2020).
35. P. Riva-Posse, K. S. Choi, P. E. Holtzheimer, C. C. McIntyre, R. E. Gross, A. Chaturvedi, A. L. Crowell, S. J. Garlow, J. K. Rajendra, H. S. Mayberg, Defining critical white matter pathways mediating successful subcallosal cingulate deep brain stimulation for treatment-resistant depression. *Biol. Psychiatry* **76**, 963–969 (2014).
36. P. Riva-Posse, K. S. Choi, P. E. Holtzheimer, A. L. Crowell, S. J. Garlow, J. K. Rajendra, C. C. McIntyre, R. E. Gross, H. S. Mayberg, A connectomic approach for subcallosal cingulate deep brain stimulation surgery: Prospective targeting in treatment-resistant depression. *Mol. Psychiatry* **23**, 843–849 (2018).
37. C. Hamani, B. Davidson, A. Levitt, Y. Meng, F. Corchs, A. Abrahao, J. S. Rabin, P. Giacobbe, N. Lipsman, Deep brain stimulation for treatment resistant post-traumatic stress disorder: A feasibility study. *Biol. Psychiatry* **1**, S0006-3223(20)31624-3, (2020).
38. P. E. Holtzheimer, M. M. Husain, S. H. Lisanby, S. F. Taylor, L. A. Whitworth, S. McClintock, K. V. Slavin, J. Berman, G. M. McKhann, P. G. Patil, B. R. Rittberg, A. Abosch, A. K. Pandurangi, K. L. Holloway, R. W. Lam, C. R. Honey, J. S. Neimat, J. M. Henderson, C. DeBattista, A. J. Rothschild, J. G. Pilitsis, R. T. Espinoza, G. Petrides, A. Y. Mogilner, K. Matthews, D. Peichel, R. E. Gross, C. Hamani, A. M. Lozano, H. S. Mayberg, Subcallosal cingulate deep brain stimulation for treatment-resistant depression: A multisite, randomised, sham-controlled trial. *Lancet Psychiatry* **4**, 839–849 (2017).
39. C. Hamani, H. Mayberg, S. Stone, A. Laxton, S. Haber, A. M. Lozano, The subcallosal cingulate gyrus in the context of major depression. *Biol. Psychiatry* **69**, 301–308 (2011).
40. R. Reznikov, C. Hamani, Posttraumatic stress disorder: Perspectives for the use of deep brain stimulation. *Neuromodulation* **20**, 7–14 (2017).
41. M. F. Marin, R. G. Zsido, H. Song, N. B. Lasko, W. D. S. Killgore, S. L. Rauch, N. M. Simon, M. R. Milad, Skin conductance responses and neural activations during fear conditioning and extinction recall across anxiety disorders. *JAMA Psychiat.* **74**, 622–631 (2017).
42. N. C. McLaughlin, D. Strong, A. Abrantes, S. Garnaat, A. Cerny, C. O'Connell, R. Fadok, C. Spofford, S. A. Rasmussen, M. R. Milad, B. D. Greenberg, Extinction retention and fear renewal in a lifetime obsessive-compulsive disorder sample. *Behav. Brain Res.* **280**, 72–77 (2015).
43. R. J. Koek, J. P. Langevin, S. E. Krahl, H. J. Kosoyan, H. N. Schwartz, J. W. Chen, R. Melrose, M. J. Mandelkern, D. Sultzer, Deep brain stimulation of the basolateral amygdala

for treatment-refractory combat post-traumatic stress disorder (PTSD): Study protocol for a pilot randomized controlled trial with blinded, staggered onset of stimulation. *Trials* **15**, 356 (2014).
44. Z. Solomon, D. Horesh, T. Ein-Dor, A. Ohry, Predictors of PTSD trajectories following captivity: A 35-year longitudinal study. *Psychiatry Res.* **199**, 188–194 (2012).
45. N. Morina, J. M. Wicherts, J. Lobbrecht, S. Priebe, Remission from post-traumatic stress disorder in adults: A systematic review and meta-analysis of long term outcome studies. *Clin. Psychol. Rev.* **34**, 249–255 (2014).
46. C. Hamani, J. N. Nobrega, Preclinical studies modeling deep brain stimulation for depression. *Biol. Psychiatry* **72**, 916–923 (2012).
47. C. Hamani, Y. Temel, Deep brain stimulation for psychiatric disease: Contributions and validity of animal models. *Sci. Transl. Med.* **4**, 142rv148 (2012).
48. D. C. O'Doherty, K. M. Chitty, S. Saddiqui, M. R. Bennett, J. Lagopoulos, A systematic review and meta-analysis of magnetic resonance imaging measurement of structural volumes in posttraumatic stress disorder. *Psychiatry Res.* **232**, 1–33 (2015).
49. F. Ahmed-Leitao, G. Spies, L. van den Heuvel, S. Seedat, Hippocampal and amygdala volumes in adults with posttraumatic stress disorder secondary to childhood abuse or maltreatment: A systematic review. *Psychiatry Res. Neuroimaging* **256**, 33–43 (2016).
50. V. Francati, E. Vermetten, J. D. Bremner, Functional neuroimaging studies in posttraumatic stress disorder: Review of current methods and findings. *Depress. Anxiety* **24**, 202–218 (2007).
51. E. A. Stark, C. E. Parsons, T. J. Van Hartevelt, M. Charquero-Ballester, H. McManners, A. Ehlers, A. Stein, M. L. Kringelbach, Post-traumatic stress influences the brain even in the absence of symptoms: A systematic, quantitative meta-analysis of neuroimaging studies. *Neurosci. Biobehav. Rev.* **56**, 207–221 (2015).
52. A. Del Casale, S. Ferracuti, A. S. Barbetti, P. Bargagna, P. Zega, A. Iannuccelli, F. Caggese, T. Zoppi, G. P. De Luca, G. Parmigiani, I. Berardelli, M. Pompili, Grey matter volume reductions of the left hippocampus and amygdala in PTSD: A coordinate-based meta-analysis of magnetic resonance imaging studies. *Neuropsychobiology* **81**, 257–264 (2022).
53. L. Meng, J. Jiang, C. Jin, J. Liu, Y. Zhao, W. Wang, K. Li, Q. Gong, Trauma-specific grey matter alterations in PTSD. *Sci. Rep.* **6**, 33748 (2016).
54. A. Starcevic, S. Postic, Z. Radojicic, B. Starcevic, S. Milovanovic, A. Ilankovic, I. Dimitrijevic, A. Damjanovic, M. Aksic, V. Radonjic, Volumetric analysis of amygdala, hippocampus, and prefrontal cortex in therapy-naive PTSD participants. *Biomed. Res. Int.* **2014**, 968495 (2014).
55. A. Badura-Brack, T. J. McDermott, E. Heinrichs-Graham, T. J. Ryan, M. M. Khanna, D. S. Pine, Y. Bar-Haim, T. W. Wilson, Veterans with PTSD demonstrate amygdala hyperactivity while viewing threatening faces: A MEG study. *Biol. Psychol.* **132**, 228–232 (2018).
56. G. A. Fonzo, M. S. Goodkind, D. J. Oathes, Y. V. Zaiko, M. Harvey, K. K. Peng, M. E. Weiss, A. L. Thompson, S. E. Zack, S. E. Lindley, B. A. Arnow, B. Jo, B. O. Rothbaum, A. Etkin, Amygdala and insula connectivity changes following psychotherapy for posttraumatic stress disorder: A randomized clinical trial. *Biol. Psychiatry* **89**, 857–867 (2021).
57. M. R. Milad, R. K. Pitman, C. B. Ellis, A. L. Gold, L. M. Shin, N. B. Lasko, M. A. Zeidan, K. Handwerger, S. P. Orr, S. L. Rauch, Neurobiological basis of failure to recall extinction memory in posttraumatic stress disorder. *Biol. Psychiatry* **66**, 1075–1082 (2009).
58. T. Raij, A. Nummenmaa, M. F. Marin, D. Porter, S. Furtak, K. Setsompop, M. R. Milad, Prefrontal cortex stimulation enhances fear extinction memory in humans. *Biol. Psychiatry* **84**, 129–137 (2018).
59. C. A. Rabinak, S. Mori, M. Lyons, M. R. Milad, K. L. Phan, Acquisition of CS-US contingencies during Pavlovian fear conditioning and extinction in social anxiety disorder and posttraumatic stress disorder. *J. Affect. Disord.* **207**, 76–85 (2017).
60. A. Nestgaard Rod, C. Schmidt, Complex PTSD: What is the clinical utility of the diagnosis? *Eur. J. Psychotraumatol.* **12**, 2002028 (2021).
61. C. R. Brewin, M. Cloitre, P. Hyland, M. Shevlin, A. Maercker, R. A. Bryant, A. Humayun, L. M. Jones, A. Kagee, C. Rousseau, D. Somasundaram, Y. Suzuki, S. Wessely, M. van Ommeren, G. M. Reed, A review of current evidence regarding the ICD-11 proposals for diagnosing PTSD and complex PTSD. *Clin. Psychol. Rev.* **58**, 1–15 (2017).
62. T. Karatzias, M. Cloitre, A. Maercker, E. Kazlauskas, M. Shevlin, P. Hyland, J. I. Bisson, N. P. Roberts, C. R. Brewin, PTSD and complex PTSD: ICD-11 updates on concept and measurement in the UK, USA, Germany and Lithuania. *Eur. J. Psychotraumatol.* **8**, 1418103 (2017).
63. F. C. Yeh, V. J. Wedeen, W. Y. Tseng, Generalized q-sampling imaging. *IEEE Trans. Med. Imaging* **29**, 1626–1635 (2010).
64. C. Hamani, H. Mayberg, B. Snyder, P. Giacobbe, S. Kennedy, A. M. Lozano, Deep brain stimulation of the subcallosal cingulate gyrus for depression: Anatomical location of active contacts in clinical responders and a suggested guideline for targeting. *J. Neurosurg.* **111**, 1209–1215 (2009).
65. A. Horn, N. Li, T. A. Dembek, A. Kappel, C. Boulay, S. Ewert, A. Tietze, A. Husch, T. Perera, W. J. Neumann, M. Reisert, H. Si, R. Oostenveld, C. Rorden, F. C. Yeh, Q. Fang, T. M. Herrington, J. Vorwerk, A. A. Kuhn, Lead-DBS v2: Towards a comprehensive pipeline for deep brain stimulation imaging. *Neuroimage* **184**, 293–316 (2019).
66. B. B. Avants, N. J. Tustison, G. Song, P. A. Cook, A. Klein, J. C. Gee, A reproducible evaluation of ANTs similarity metric performance in brain image registration. *Neuroimage* **54**, 2033–2044 (2011).
67. D. N. Greve, C. Svarer, P. M. Fisher, L. Feng, A. E. Hansen, W. Baare, B. Rosen, B. Fischl, G. M. Knudsen, Cortical surface-based analysis reduces bias and variance in kinetic modeling of brain PET data. *Neuroimage* **92**, 225–236 (2014).
68. D. N. Greve, D. H. Salat, S. L. Bowen, D. Izquierdo-Garcia, A. P. Schultz, C. Catana, J. A. Becker, C. Svarer, G. M. Knudsen, R. A. Sperling, K. A. Johnson, Different partial volume correction methods lead to different conclusions: An (18)F-FDG-PET study of aging. *Neuroimage* **132**, 334–343 (2016).
69. M. Jenkinson, C. F. Beckmann, T. E. Behrens, M. W. Woolrich, S. M. Smith, FSL. *Neuroimage* **62**, 782–790 (2012).

Acknowledgments: We thank Anusha Baskaran for the technical support. **Funding:** This investigator-initiated study was supported with funds from the DANA foundation, Veteran Affairs Canada, and the Harquail Centre for Neuromodulation. **Author contributions:** C.H., B.D., F.C., P.G., and N.L. contributed to the study conceptualization and design. C.H., B.D., A.A., S.M.N., J.S.R., A.N., L.P., M.G., O.T., P.G., and N.L. participated in data collection. A.L. provided technical expertise. C.H., B.D., J.S.R., P.G., and N.L. had full access to the data and contributed to the analysis and interpretation. All authors critically revised the paper. **Competing interests:** The authors declare that they have no competing interests. **Data and materials availability:** All data needed to evaluate the conclusions in the paper are present in the paper and/or the Supplementary Materials.

Submitted 13 May 2022
Accepted 17 October 2022
Published 2 December 2022
10.1126/sciadv.adc9970
