# Supplementary material for: Deep brain stimulation of the subgenual cingulum and uncinate fasciculus for the treatment of posttraumatic stress disorder
Source: Sci Adv. 2022 Dec 2;8(48):eadc9970. doi: 10.1126/sciadv.adc9970 (PMC10936049; doi:10.1126/sciadv.adc9970)
Supplement: Supplementary file 2 — Figs. S1 and S2 Tables S1 to S4 [file sciadv.adc9970_sm.v2.pdf]

CORRECTED 26 JANUARY 2024; SEE ERRATUM

Science Advances
AAAS

Supplementary Materials for

Deep brain stimulation of the subgenual cingulum and uncinate fasciculus for the treatment of posttraumatic stress disorder

Clement Hamani *et al.*

Corresponding author: clement.hamani@sunnybrook.ca

Sci. Adv. **8**, eadc9970 (2022)
DOI: 10.1126/sciadv.adc9970

This PDF file includes:

Figs. S1 and S2
Tables S1 to S4

CORRECTED 26 JANUARY 2024; SEE ERRATUM

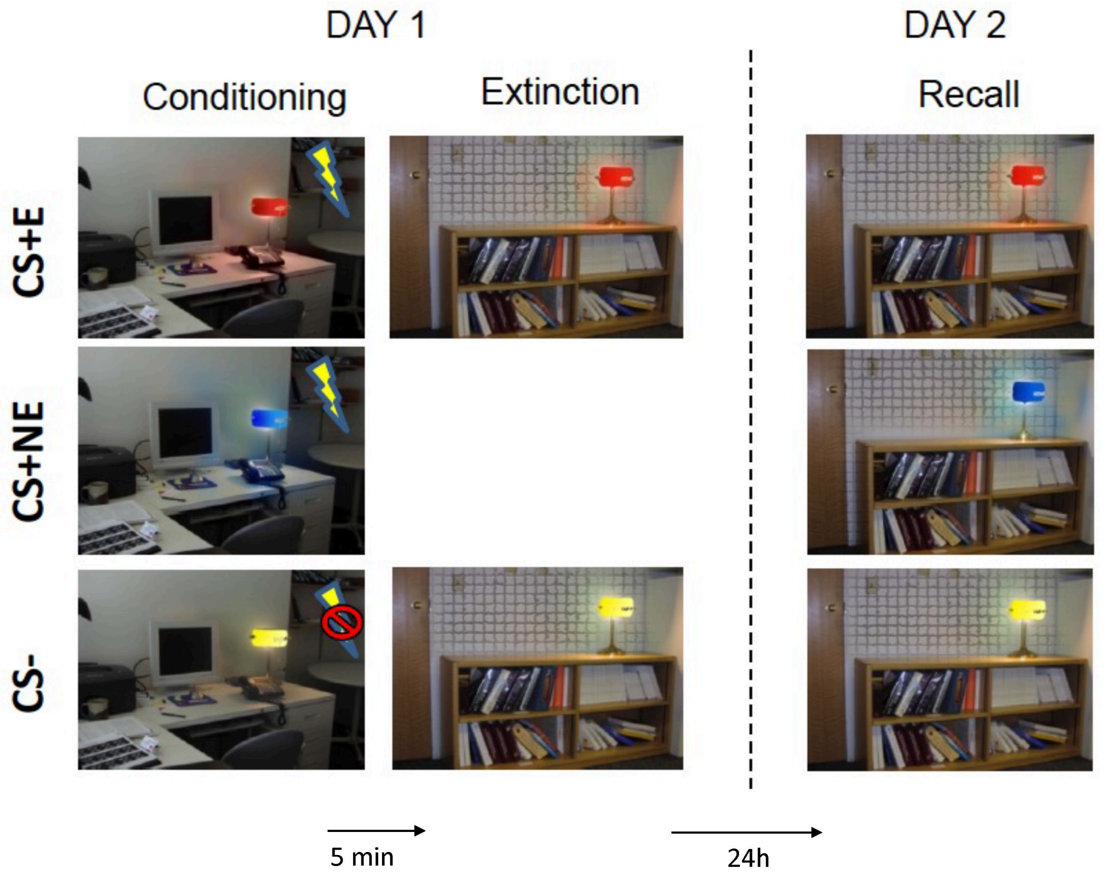

Supplementary Figure 1. Schematic representation of the psychophysiological experiments used in our trial. During fear conditioning, images of an office with a table lamp were presented to participants. Thereafter images with the lamp shining a red or a blue light were presented eight times for 6 seconds. In 5 of these trials image presentation was followed by the delivery of a 0.5 second electric shock (CS+; thunderbolt). The third colored lamp (yellow) was presented 16 times with no associated shock (CS-). Five minutes later, subjects underwent a fear extinction phase, during which they looked at images containing similar lamps in a different context (a library instead of an office). The CS+ red light was presented 16 times with no electric shock pairing. Images were intermixed with 16 presentations of the CS- (yellow light; 6 seconds per image). On

the following day, participants were exposed to extinction memory recall testing. Both extinguished and non-extinguished CS+ were presented 8 times each, along with 16 presentations of the CS-. No shocks were delivered during recall. Figure adapted and reprinted from reference (*42;* Marin et al, 2017 JAMA Psychiatry 74, 622-631) with permission from the American Medical Association.

CORRECTED 26 JANUARY 2024; SEE ERRATUM

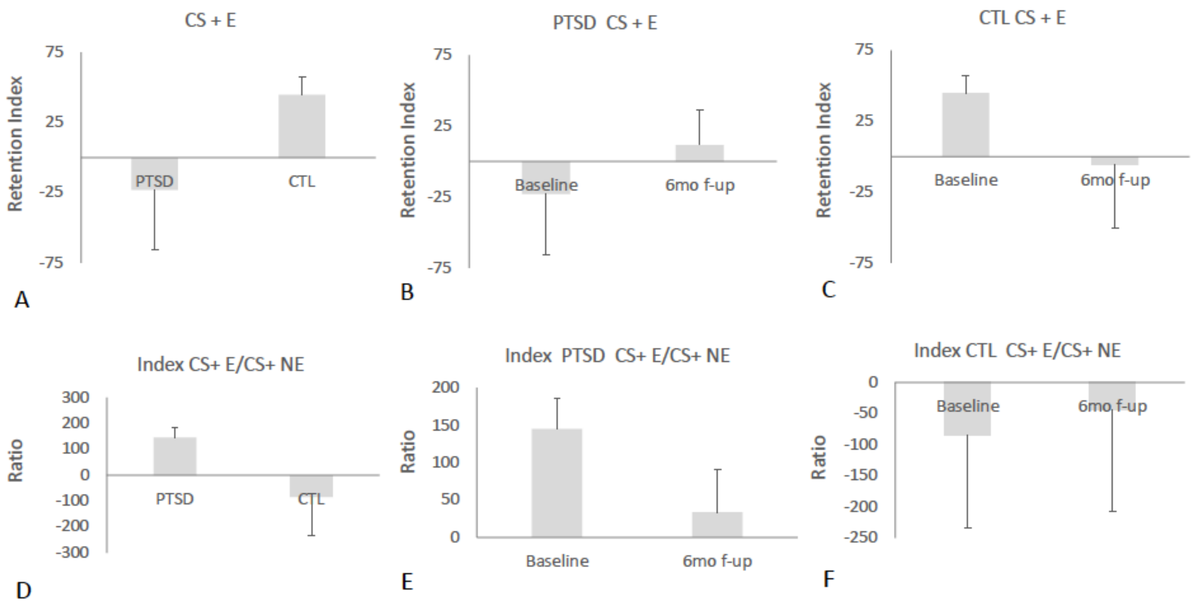

Supplementary Figure 2. Indices to decrease the variance in skin conductance responses (SCR) during recall. The extinction retention index (ERI) was calculated according to the formula 100 - ([recall value/ acquisition value] X 100). (A) While no comparison was found to be significant, patients with posttraumatic stress disorder (PTSD) had lower values than controls (CTL), possibly denoting an extinction deficit. (B) During postoperative follow-up visits, the extinction retention index in PTSD patients was found to be higher than at baseline, which is in line with the reduced SCR measured during recall. Results in controls were more difficult to interpret (C), as follow-up acquisition and recall SDR levels were very close to zero. The ratio between the extinction retention index recorded during extinguished (CS+ E) and non-extinguished (CS+ NE) stimuli was calculated as: CS+E / CS+NE*100. While no significant differences were found between PTSD patients and controls in the preoperative period (D) or during follow-up assessments (E, F), we found that controls had a higher retention of CS+E relative to CS+NE, whereas PTSD patients had the opposite trend. Of note, PTSD patients had a substantial

decrease in this ratio compared to baseline, denoting a possible improvement in the postoperative period (E). This trend was not observed in controls (F).

Suppl Table 1. Individual Clinician-Administered Posttraumatic Stress Disorder Scale (CAPS) scores

	Baseline	wk 2	3mo	6mo	% improvement 6mo	Responder
Patient 1	56	52	1	0	100	Yes
Patient 2	63	2	16	16	74.6	Yes
Patient 3	57	59	42	37	35.1	Partial
Patient 4	66	57	48	56	15.2	No

Abbreviations: mo- month; wk- week

CORRECTED 26 JANUARY 2024; SEE ERRATUM

Suppl Table 2. Individual Hamilton Depression Rating Scale 17 items (HAMD) scores

	Baseline	wk 2	3mo	6mo	% improvement 6mo	Responder
Patient 1	26	23	7	12	53.8	Yes
Patient 2	22	0	6	5	77.3	Yes
Patient 3	32	24	26	23	28.1	Partial
Patient 4	32	34	24	35	-9.4	No

Abbreviations: mo- month; wk- week

CORRECTED 26 JANUARY 2024; SEE ERRATUM

Suppl Table 3. Neuropsychological test performance at baseline and 6 months following DBS

Cognitive test	n	Baseline	6 mo	*P*
*WTAR[d]	4	118.00 (2.25)	–	–
CVLT-II: total[a]	4	57.00 (5.75)	62.00 (13.50)	0.25
CVLT-II: delayed free recall[b]	4	0.75 (1.00)	0.50 (0.38)	1.00
Oral SDMT[a]	4	49.00 (15.75)	50.50 (14.75)	0.88
D-KEFS Sorting Task: Correct number of sorts[c]	3	13.00 (0.50)	13.00 (2.00)	1.00
D-KEFS Sorting Task: Description score[c]	3	13.00 (0.00)	13.00 (2.00)	1.00
BVMT-R: Immediate recall[a]	3	45.00 (13.50)	63.00 (7.50)	0.25
BVMT-R: Delayed recall[a]	3	61.00 (9.50)	55.00 (4.00)	1.00
D-KEFS Phonemic Fluency[c]	3	14.00 (3.50)	12.00 (3.00)	0.75
Category Fluency (Animals)[a]	3	63.00 (5.50)	58.00 (7.50)	0.75
FRsBE Apathy Scale[a]	4	104.50 (10.25)	91.50 (10.25)	0.13
FRsBE Dysexecutive Scale[a]	4	68.00 (12.75)	61.00 (7.50)	0.18
FRsBE Disinhibition Scale[a]	4	69.00 (10.00)	61.00 (10.75)	0.25
FRsBE Total[a]	4	83.00 (15.25)	74.00 (9.75)	0.10

Abbreviations: BVMT-R, Brief Visuospatial Memory Test-Revised; CVLT-II, California Verbal Learning Test, Second Edition; D-KEFS, Delis-Kaplan Executive Function System; FRsBE, Frontal Systems Behavior Scale (Self-version); SDMT Symbol Digit Modalities Test; WTAR, Wechsler Test of Adult Reading.
Median scores and interquartile ranges of are presented.

[a] t-score (mean of 50, SD of 10); [b]Z-score (mean of 0, SD of 1); [c]Scaled score (mean of 10, SD of 3); [d]Standard score (mean of 100, SD of 15).
*WTAR was only administered at baseline to estimate premorbid intellectual functioning.
All scores were standardized with published normative data.
P values reflect the results of a Wilcoxon signed rank test.

CORRECTED 26 JANUARY 2024; SEE ERRATUM

Suppl Table 4. Metabolic activity as measured with fluorodeoxyglucose positron emission tomography

	Baseline	Postoperative	Change (%)	p-FDR
**Right**				
Amygdala	1.2 ± 0.1	1.3 (0.07	+ 4.1	0.71
Hippocampus	1.3 ± 0.1	1.3 (0.0	+ 0.9	0.71
Accumbens	1.9 ± 0.6	1.7 (0.2	- 14.7	0.65
ACC	1.8 ± 0.3	1.9 (0.3	+ 6.5	**0.002***
Medial OFC	2.4 ± 0.3	2.4 (0.2	+ 1.2	0.71
Frontal Pole	3.1 ± 0.2	3.2 (0.2	+ 2.2	0.71
**Left**				
Amygdala	1.1 ± 0.1	1.2 ± 0.1	+ 5.0	**0.03***
Hippocampus	1.3 ± 0.1	1.3 ± 0.3	+ 3.5	0.10
Accumbens	2.2 ± 0.7	2.0 ± 0.3	- 10.0	0.79
ACC	2.1 ± 0.4	1.9 ± 0.3	- 5.2	0.10
Medial OFC	2.4 ± 0.3	2.5 ± 0.3	+ 1.5	0.83
Frontal Pole	3.1 ± 0.5	3.1 ± 0.5	+ 0.8	0.88

Abbreviations: ACC- anterior cingulate gyrus; FRD- false discovery ratio; OFC- orbitofrontal cortex. Data represents mean ± standard deviation. * statistically significant.
